# Nondestructive, real-time determination and visualization of cellulose, hemicellulose and lignin by luminescent oligothiophenes

**DOI:** 10.1038/srep35578

**Published:** 2016-10-19

**Authors:** Ferdinand X. Choong, Marcus Bäck, Svava E. Steiner, Keira Melican, K. Peter R. Nilsson, Ulrica Edlund, Agneta Richter-Dahlfors

**Affiliations:** 1 Swedish Medical Nanoscience Center, Department of Neuroscience, Karolinska Institutet, Stockholm, SE-171 77, Sweden; 2 Division of Chemistry, Department of Physics, Chemistry and Biology, Linköping University, Linköping, SE-581 83, Sweden; 3 Fiber and Polymer Technology, KTH Royal Institute of Technology, Stockholm, SE-100 44, Sweden

## Abstract

Enabling technologies for efficient use of the bio-based feedstock are crucial to the replacement of oil-based products. We investigated the feasibility of luminescent conjugated oligothiophenes (LCOs) for non-destructive, rapid detection and quality assessment of lignocellulosic components in complex biomass matrices. A cationic pentameric oligothiophene denoted p-HTEA (pentamer hydrogen thiophene ethyl amine) showed unique binding affinities to cellulose, lignin, hemicelluloses, and cellulose nanofibrils in crystal, liquid and paper form. We exploited this finding using spectrofluorometric methods and fluorescence confocal laser scanning microscopy, for sensitive, simultaneous determination of the structural and compositional complexities of native lignocellulosic biomass. With exceptional photostability, p-HTEA is also demonstrated as a dynamic sensor for real-time monitoring of enzymatic cellulose degradation in cellulolysis. These results demonstrate the use of p-HTEA as a non-destructive tool for the determination of cellulose, hemicellulose and lignin in complex biomass matrices, thereby aiding in the optimization of biomass-converting technologies.

Being a major component of all plant matter, cellulose represents one of the most abundant polymers in nature. Due to the relative resistance to chemical changes, low solubility and considerable tensile strength, cellulose materials have a wide range of industrial applications, including textiles, paper, membranes, and packaging. Separation into nanoscale fibrils (nanocellulose) has widened the application areas even further^1^,^2^, exemplified by nanoparticles and nanocomposites. Cellulose thus represents an omnipresent substance of immense economic importance^3^. With the increasing global awareness of sustainability issues, cellulosic biomass is being revisited as a robust future alternative to the conventional fossil feedstock, viable for biorefining, where the generated fractions can be converted into bulk chemicals, materials and biofuel^4^,^5^,^6^. Optimizing biomass processing requires, however, rapid and easy methods for determination of the chemical composition, which varies depending on the plant species, the location of its growth, and its physiological age.

Lignocellulosic biomass is composed of carbohydrate polymers, primarily cellulose and hemicellulose, co-existing in complex matrices with the highly aromatic biopolymer lignin^7^. The cellulose polysaccharide is composed of chains of linear β(1-4) linked D-glucose units, which associate via multiple hydrogen bonds to form microfibrils^8^. Whereas cellulose is structurally homogenous, hemicelluloses are typically heterogeneous in terms of sugar composition and branching, and lignin is a non-repetitive network. The dispersity of such macromolecular structures makes their detection and chemical identification a challenging task. Recently, a near-infrared (NIR) method for detection of lignin, hemicellulose and cellulose components in lignocellulosic matrices was reported^9^,^10^. While offering a means of non-destructive analysis, interpretation quality of the complex NIR spectra, with numerous broad and highly overlapping absorption peaks, requires reliable calibration algorithms in order to model the full array of independent variables. Also, pre-treatments may be needed to remove effects of interference factors^10^,^11^. Traditionally, wet chemistry techniques are used for composition analysis^12^,^13^,^14^,^15^. They entail indirect methods and/or technically demanding approaches, often coupled to degradation and solubilisation of the biomass via harsh pre-treatment such as alkaline or acid hydrolysis, followed by analytical techniques such as ion chromatography, nuclear magnetic resonance, mass spectrometry, and X-ray crystallography^14^,^15^,^16^,^17^. Defining the nature of the polymer from which monomers of the disintegrated sample originates can be very challenging. These laborious, time consuming, and chemical reagent consuming methods also suffer from difficulties of up-scaling, automation, integration and widespread application in large-scale industrial biomass processes, particularly when the cellulosic component is present in a complex matrix, such as the native lignocellulosic feedstock. Whereas on-line video recording systems can monitor fibre dimensions in real-time, the actual chemical composition remains unknown.

Luminescent conjugated oligothiophenes represent a class of structure-sensitive molecular chameleons, which dynamically alter their optical properties. Distortion of the flexible conjugated thiophene backbone in response to non-covalent electrostatic interactions with target molecules generates specific spectral signatures, which exhibits an ON/OFF like switching of fluorescence activation^18^,^19^,^20^. The chemical evolution of oligothiophenes, i.e. going from polydispersed materials to chemically defined molecules, have generated oligothiophene molecules that can be utilized as multimodal tools for optical detection and identification of disease-associated protein aggregates, the pathological hallmark in Alzheimer´s disease and other neurodegenerative diseases^19^,^20^. Based on the binding-induced spectroscopic signatures in the excitation and emission spectra of the oligothiophenes^20^,^21^, fluorescence microscopy and spectrophotometric recordings can be applied to optically trace protein aggregates both *in vitro* and *in vivo*^22^,^23^. Interactions between the opto-tracing oligothiophene molecule and protein aggregates characteristically lead to the flattening of the molecular backbone and more effective conjugation, causing a red-shift in fluorescence excitation and an increased fluorescence emission intensity^18^,^19^,^20^. The excitation spectrum in particular is a direct reflection of the oligothiophene backbone geometry.

We hypothesize a novel use of LCOs as fluorescent opto-tracers of polysaccharides in the lignocellulose feedstock. To our knowledge, neither conventional dyes, nor other techniques offer a simple, one-step method for real-time detection and differentiation of cellulose, hemicellulose and lignin in their native polymeric structure. To address the need for non-destructive analysis, we investigate the potential of a subset of oligothiophenes for specific, rapid and direct spectrofluorometric detection and profiling of lignocellulosic biomass. We demonstrate the identification and characterization of the oligothiophene named p-HTEA (pentamer hydrogen thiophene ethyl amine) as a novel probe to detect the structure of cellulose in numerous forms and to assess cellulose purity in conjunction with lignin and hemicellulose detection. We also validate real-life applications in both sulphite and kraft pulping, defining the chemical composition of the pulp^24^,^25^. Moreover, we demonstrate how p-HTEA can be applied to dynamically monitor enzymatic degradation of cellulose in real-time, a method highly relevant in the production of ethanol-based biofuel.

## Results

### Cellulose detection by p-HTEA

**Figure 1:**
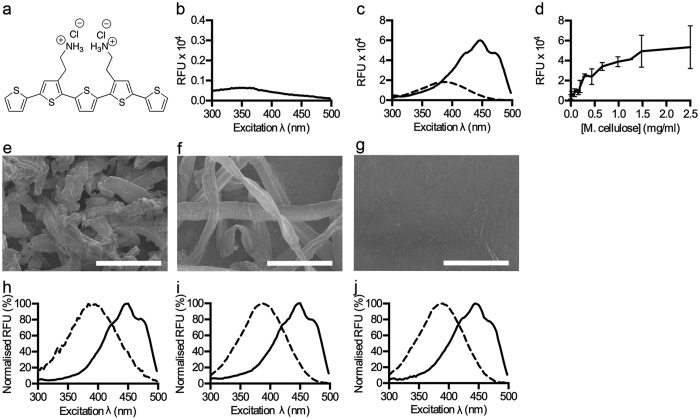
Optical detection of cellulose and cellulose nanofibrils by p-HTEA. (**a**) Structure of p-HTEA. (**b,c**) Excitation spectra of cellulose in the absence and presence of p-HTEA. Spectra were collected at λ_Ex_ = 300–500 nm, and λ_Em_ = 545 nm of (**b**) M. cellulose (5 mg/ml), (**c**) p-HTEA (dashed), and M. cellulose mixed with p-HTEA (solid). (**d**) Correlation of the fluorescence signal (RFU) from p-HTEA + M. cellulose, recorded at λ_Ex_ = 447 nm, λ_Em_ = 545 nm, to varying M. cellulose concentrations. Error bars = SD, n = 3. (**e–g**) Scanning electron micrographs of cellulosic materials. (**e**) M. cellulose (**f**) pulp cellulose and (**g**) cellulose nanofibrils. Scale bars (**e,f**) = 100 μm, (**g**) = 10 μm. (**h–j**) Normalized excitation spectra of p-HTEA bound to cellulosic materials. Spectra were collected at λ_Ex_ = 300–500 nm and λ_Em_ = 545 nm of p-HTEA (dashed) or p-HTEA mixed with (**h**) M. cellulose, (**i**) pulp cellulose, (**j**) cellulose nanofibrils (solid). Normalized relative fluorescence units (RFU) = each data point represented as a percentage of the largest emitted fluorescence of the excitation spectra.

Starting from a panel of pentameric oligothiophenes with specific side chain functionalities, we selected p-HTEA^26^,^27^, a pentameric oligothiophene functionalized with two ethylamine groups, as a potential candidate for the targeted detection of cellulose (Fig. 1a). Possible interactions between p-HTEA and microcrystalline cellulose (M. cellulose) were evaluated by collecting the fluorescence excitation spectrum of M. cellulose with and without p-HTEA using a spectrophotometer. Binding of p-HTEA to M. cellulose resulted in a specific interaction, leading to increased fluorescence, observed as relative fluorescence units (RFU), in the excitation spectrum compared to the native spectrum of M. cellulose (Fig. 1b,c). A red-shift in the wavelength of excitation maximum (λ_max_) to 444 nm when compared to the spectrum of the unbound p-HTEA (λ_max_ = 386 nm) was also observed (Fig. 1c). Together with the two characteristic shoulders on each side of the major excitation peak, these changes represent a unique optical signature of p-HTEA bound to M. cellulose. The sensitivity of p-HTEA for cellulose was further profiled by analysing the excitation spectrum at different concentrations of M. cellulose. Increasing concentrations resulted in increasing RFU up to 2.5 mg/ml at which point the optics of the system limited further signal increase due to sample density (Fig. 1d).

Cellulose in both its natural and modified forms vary drastically in dimensions, purity, and morphology. The latter is clearly shown by scanning electron microscopy. Crystals of M. cellulose and fibres of bleached pulp (pulp cellulose) are both observable at a 10 times lower magnification than nanocellulose fibres, which in this study are fibrils sub-μm in thickness (Fig. 1e–g). Since the varying structures can lead to non-homogenous exposure of hydrophilic and hydrophobic surface areas, or the exposure of functional groups acting as binding sites for dyes and enzymes^28^,^29^, we analysed whether excitation spectra from p-HTEA bound to M. cellulose, pulp cellulose and cellulose nanofibrils were affected. All spectra exhibited the characteristic red-shift of λ_max_ to 444 nm and the two shoulders, indicative of p-HTEA-cellulose interaction (Fig. 1h–j). An increase in RFU was also observed (Supplementary Fig. 1), indicating that the better resolved emission from p-HTEA bound to cellulose might be induced from p-HTEA-molecules released from aggregation upon interaction with cellulose. The identical spectral profiles of bound p-HTEA regardless of the cellulose dimension suggests a direct binding of p-HTEA to the β(1-4) D-glucose chain or a common structure in the tested cellulose morphologies.

### Binding of p-HTEA to lignin compared to cellulose

**Figure 2:**
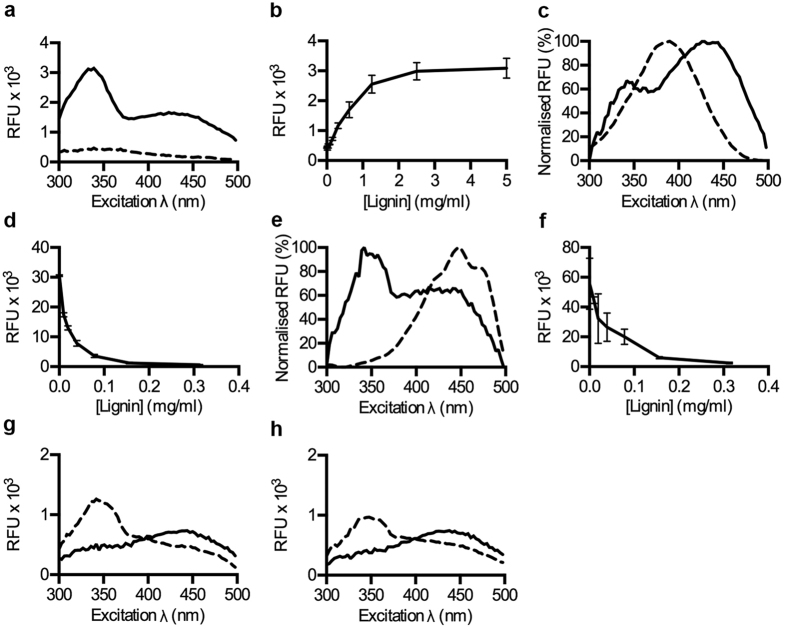
Binding of p-HTEA to lignin enables optical identification of lignin in mixed samples. **(a**) Excitation spectra of lignin in ^D^H_2_O (solid) and ^D^H_2_O alone (dashed) collected at λ_Ex_ = 300–500 nm, λ_Em_ = 545 nm. (**b**) Correlation between intrinsic fluorescence signals at λ_Ex_ 339 nm, λ_Em_ 545 nm, and lignin concentration. (**c**) Normalized excitation spectra of p-HTEA in the absence (dashed) and presence of lignin (solid). (**d**) Correlation between p-HTEA fluorescence signals at λ_max-p-HTEA_ (λ_Ex_ 387 nm, λ_Em_ 545 nm) and lignin concentration. (**e**) Normalized excitation spectra of p-HTEA bound to cellulose before (dashed) and after (solid) addition of lignin. (**f**) Correlation between fluorescence signals recorded at λ_max-cellulose_ (λ_Ex_ 444 nm, λ_Em_ 545 nm) of p-HTEA pre-bound to cellulose with different added concentrations of lignin. (**g**) Excitation spectra of p-HTEA + lignin (dashed) and p-HTEA + lignin + cellulose (solid). (**h**) Excitation spectra of intrinsic fluorescence of lignin (dashed) and lignin + cellulose (solid). All panels show the mean of triplicates from one out of three experimental repeats.

Delignification is a major process in isolation and recovery of materials from lignocellulosic biomass. With the ongoing discovery of new applications for both cellulose and lignin, rapid methods for quality control are essential. Lignin is an extensively branched polymer composed of aromatic building blocks and is therefore inherently fluorescent. Analysis of its fluorescence excitation spectrum shows an excitation peak at 339 nm and a broad shoulder centred at 425 nm (Fig. 2a). When comparing the RFU of lignin at different concentrations, an increasing trend was observed, which reached a plateau above 2.5 mg/ml of lignin (Fig. 2b). This suggests that at high concentrations, self-quenching of fluorescence may occur due to interactions between the individual lignin polymers. Alternatively, the system may have reached its optical limit of detection. Addition of p-HTEA to lignin and subsequent analysis of the excitation spectrum revealed a shift in λ_max_ to 425 nm, compared to 386 nm of p-HTEA in the absence of lignin (Fig. 2c). A dramatic decrease in total RFU as compared to unbound probe was also observed (Supplementary Fig. 2a). Comparison of the RFU generated by p-HTEA in the presence of increasing lignin concentrations revealed a quenching of the fluorescence from the dye at lignin concentrations as low as 0.2 mg/ml (Fig. 2d). Collectively, this suggests a rather high affinity of p-HTEA for lignin, and that binding severely quenches the p-HTEA fluorescence. The strong binding between lignin and p-HTEA is presumably mediated by interactions between the aromatic building blocks in lignin and the thiophene backbone, since several pentameric oligothiophenes with other side chain functionalities (p-HTAA, p-FTAA and p-HTIm) also display decreased emission when mixed with lignin (Supplementary Fig. 2b–d).

We next analysed the affinity of p-HTEA to lignin compared to cellulose. Addition of lignin to a mixture of p-HTEA prebound to M. cellulose demonstrated a shift in the excitation spectra from the p-HTEA/cellulose signature, with a λ_max_ of 444 nm, to the λ_max_ of 339 nm of lignin alone (Fig. 2e). This suggests that p-HTEA has stronger affinity for lignin than for M. cellulose. In terms of RFU, addition of lignin again abrogated the p-HTEA/cellulose specific signal (Supplementary Fig. 2e). Applying increasing concentrations of lignin to prebound p-HTEA - M. cellulose mixtures again highlighted the strong competitive binding of lignin to p-HTEA with effective quenching at lignin concentrations as low as <0.3 mg/ml. (Fig. 2f). In the reverse experiment, addition of M. cellulose to premixed samples of p-HTEA and lignin did not result in the cellulose specific p-HTEA excitation profile (Fig. 2g, Supplementary Fig. 2f). The small changes in the excitation spectrum were rather due to lignin - cellulose interactions, as a similar result was obtained in the absence of p-HTEA (Fig. 2h, Supplementary Fig. 2g). Overall, we conclude that the presence of even minor amounts of contaminating lignin can be detected via a quenching of the RFU of the p-HTEA/M. cellulose signal as well as by detection of the shift in λ_max_ from the p-HTEA/cellulose specific 444 nm to 339 nm indicative of lignin. In contrast to gravimetric methods, which often overestimate lignin content due to the fibre’s acid insoluble molecules^14^, p-HTEA-based opto-tracing methods can be utilized as sensitive tests of the efficiency of delignification in lignocellulosic biomass.

### Probing hemicelluloses

**Figure 3:**
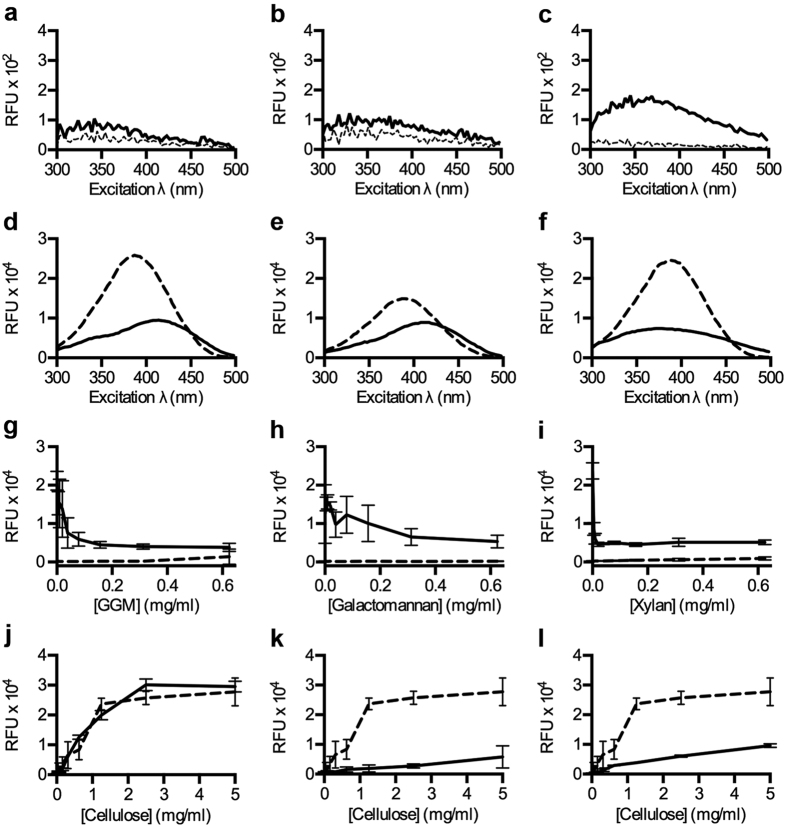
p-HTEA-based optical identification of hemicellulose in mixed samples. (**a–c**) Excitation spectra of (**a**) *O-*acetylgalactoglucomannan (solid), (**b**) galactomannan (solid) and (**c**) xylan (solid) using ^D^H_2_O (dashed) as a control. (**d–f**) Excitation spectra of p-HTEA bound to (**d**) *O-*acetylgalactoglucomannan (solid), (**e**) galactomannan (solid) and (**f**) xylan (solid) and from p-HTEA alone (dashed). (**g–i**) Correlation of the signal at λ_max_ in (d–f, solid lines) with increasing concentrations of each hemicellulose in the presence (solid) and absence (dashed) of p-HTEA. (**g**) λ_max p-HTEA-*O*-acetylgalactoglucomannan_ (λ_Ex_ 414 nm, λ_Em_ 545 nm) correlated to the concentration of *O-*acetylgalactoglucomannan (GGM), (**h**) λ_max p-HTEA-galactomannan_ (λ_Ex_ 414 nm, λ_Em_ 545 nm) to the concentration of galactomannan, and (**i**) λ_max p-HTEA-xylan_ (λ_Ex_ 375 nm, λ_Em_ 545 nm) to the concentration of xylan. (**j–l**) RFU (λ_max p-HTEA-cellulose,_ λ_Ex_ 444 nm, λ_Em_ 545 nm) from p-HTEA with increasing cellulose concentrations in the presence (solid line) and absence (dashed line) of (**j**) *O-*acetylgalactoglucomannan, (**k**) galactomannan, and (**l**) xylan.

Hemicelluloses consist of a heterogeneous group of matrix polysaccharides such as mannans and xylans^30^. To investigate the use of the opto-tracing method towards this component of the lignocellulosic biomass, we first analysed the basal fluorescence of hemicelluloses from spruce (*O-*acetylgalactoglucomannan), seeds (galactomannan) and beechwood (xylan). The excitation spectra showed a basal degree of fluorescence from all three, with xylan significantly more fluorescent than the mannans (Fig. 3a–c). We next analysed the optical profiles of p-HTEA added to individual hemicelluloses. When added to *O*-acetylgalactoglucomannan and galactomannan, quenching of the p-HTEA fluorescence was observed along with a red-shift in λ_max_, indicating linearization of the thiophene backbone upon binding to the mannans (Fig. 3d,e). In contrast, interaction of p-HTEA and xylan resulted in a blue-shift in λ_max_, indicative of a greater twist in the LCO backbone, as well as quenching of emitted fluorescence (Fig. 3f). Binding-associated quenching of p-HTEA fluorescence by hemicelluloses proved to be very sensitive. Incremental increase from 0.01–5 mg/ml of *O-*acetylgalactoglucomannan or galactomannan in the presence of p-HTEA dramatically lowered the RFU, reaching maximum effect at 0.625 mg/ml (Fig. 3g,h). Xylan exhibited an even greater aptitude for the quenching of p-HTEA with the maximum effect occurring below 0.156 mg/ml (Fig. 3i). This suggests that the different hemicelluloses possess different binding affinities for p-HTEA, which can be used to differentiate between certain hemicelluloses or different hemicellulose macromolecular architectures.

To test whether p-HTEA could be applied to identify the incomplete removal of hemicelluloses from cellulose samples, we compared the binding affinities of p-HTEA to M. cellulose and the hemicelluloses respectively. A competitive binding assay was used in which p-HTEA was premixed with the respective hemicellulose before M. cellulose was added at increasing concentrations. Increasing M. cellulose concentrations led to increasing RFU irrespective of whether the opto-tracer was premixed with *O-*acetylgalactoglucomannan (Fig. 3j). In contrast, pre-binding of p-HTEA to galactomannan or xylan significantly reduced the cellulose specific p-HTEA fluorescence (Fig. 3k,l). This indicates differential affinity of p-HTEA to the tested hemicelluloses. The increased affinity of p-HTEA to galactomannan and xylan compared to M. cellulose shows the potential of p-HTEA to be used as a sensor for hemicellulose impurities in cellulose biomasses.

### p-HTEA applied to process liquor samples and fibre suspensions

**Figure 4:**
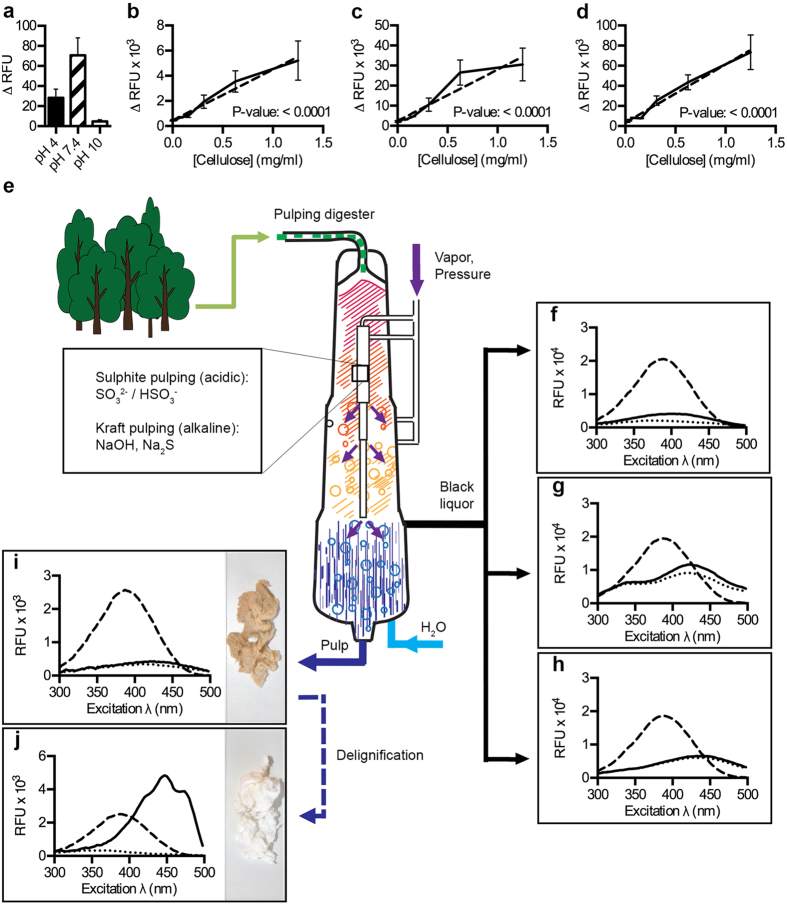
Spectral quality assessment of cellulose during the pulping process. Fluorescence signal from p-HTEA mixed with cellulose in acidic and alkaline conditions. Spectra were recorded at λ_Ex_ 445 nm, λ_Em_ 530 nm. Signal (ΔRFU = RFU _Bound p-HTEA_–RFU _Unbound p-HTEA_) from (**a**) p-HTEA bound to cellulose at pH 4, pH 7.4 and pH 10. (**b–d**) Signal from p-HTEA at increasing concentrations of cellulose (solid) at (**b**) pH 4, (**c**) pH 7.4 and (**d**) pH 10. Linear regression is shown as dashed line. (**e**) Cartoon illustrating the continuous cooking process, presenting key stages for p-HTEA quality analysis of cellulose and by-products. (**f–h**) Excitation spectra of p-HTEA mixed with (**f**) process liquor (solid), (**g**) lignosulphonate (solid), and (**h**) Kraft lignin (solid), compared to spectra of p-HTEA alone (dashed), and the basal fluorescence of each sample (dotted). (**i,j**) Excitation spectra of p-HTEA mixed with (**i**) unbleached (solid) and (**j**) bleached (solid) pulp, compared to spectra of p-HTEA alone (dashed), and the basal fluorescence of each sample (dotted). Photographs show the characteristic colours of unbleached (brownish) and bleached (white) pulp.

p-HTEA binding to target polysaccharides occurs by electrostatic interactions, which potentially can be affected by solvent properties. When considering the opto-tracing method for on-line detection of lignocellulosic materials in the continuous cooking process, it has to be recognized that cellulose extraction typically occurs under acidic or alkaline conditions^31^. To analyse the functionality of p-HTEA at acidic, neutral, and alkaline conditions, we assessed the signal quality of p-HTEA interacting with a fixed M. cellulose concentration at pH 4, 7.4 and 10. Detection of the emitted fluorescence revealed highest RFU at pH 7.4 with a slightly reduced signal intensity at pH 4, and a major drop at pH 10 (Fig. 4a). To determine whether the fluorescence signal still correlated to an increase in M. cellulose despite changes in pH, RFU from p-HTEA interacting with increasing amounts of M. cellulose was recorded at pH 4, 7.4, and 10 (Fig. 4b–d). Highly linear relationships were observed at all pH, with p-values of the linear regression line in all experiments <0.0001. This suggests that the sensitivity of p-HTEA is retained across pH 4 to 10, regardless of total RFU.

The sulphite and kraft pulping processes involve the use of acidic or alkaline chemicals at high temperatures to break covalent bonds, thereby separating the components of plant cell walls resulting in delignification and concomitant de-fibration (Fig. 4e). To investigate the use of p-HTEA in analysing cellulose purity and/or the presence of lignin and hemicellulose during the extraction process, we acquired a series of authentic samples from the fibre suspension and process liquors in the pulping digester. When analysing the fluorescence spectrum of p-HTEA applied to process liquor samples recovered from hardwood pulping, p-HTEA fluorescence was quenched due to the high content of hemicelluloses and lignin (Fig. 4f). The same pattern was observed for process liquors obtained from the delignification process, in which lignin is recovered as lignosulfonate (sulphite pulping) or Kraft lignin (alkaline pulping) (Fig. 4g,h). In all cases, the high affinity of p-HTEA to lignin strongly suppressed the inherent fluorescence of the probe. Removal of the majority of non-cellulosic components results in unbleached pulp. Despite its high cellulose content, the presence of approximately 4% lignin as determined by the pulp’s kappa number^32^,^33^ of 27.9, meant that the lignin associated quenching was still the predominant profile (Fig. 4i). Following further treatment, bleached pulp is obtained. With a kappa number <2, indicating very low amounts of lignin contamination, the unique p-HTEA-cellulose spectral signature was identified (Fig. 4j). These experiments demonstrate the suitability of p-HTEA as a sensitive opto-tracer for detection of cellulose purity in multi-component matrices.

### Fluorescence imaging and optical signatures of cellulose-derived materials

**Figure 5:**
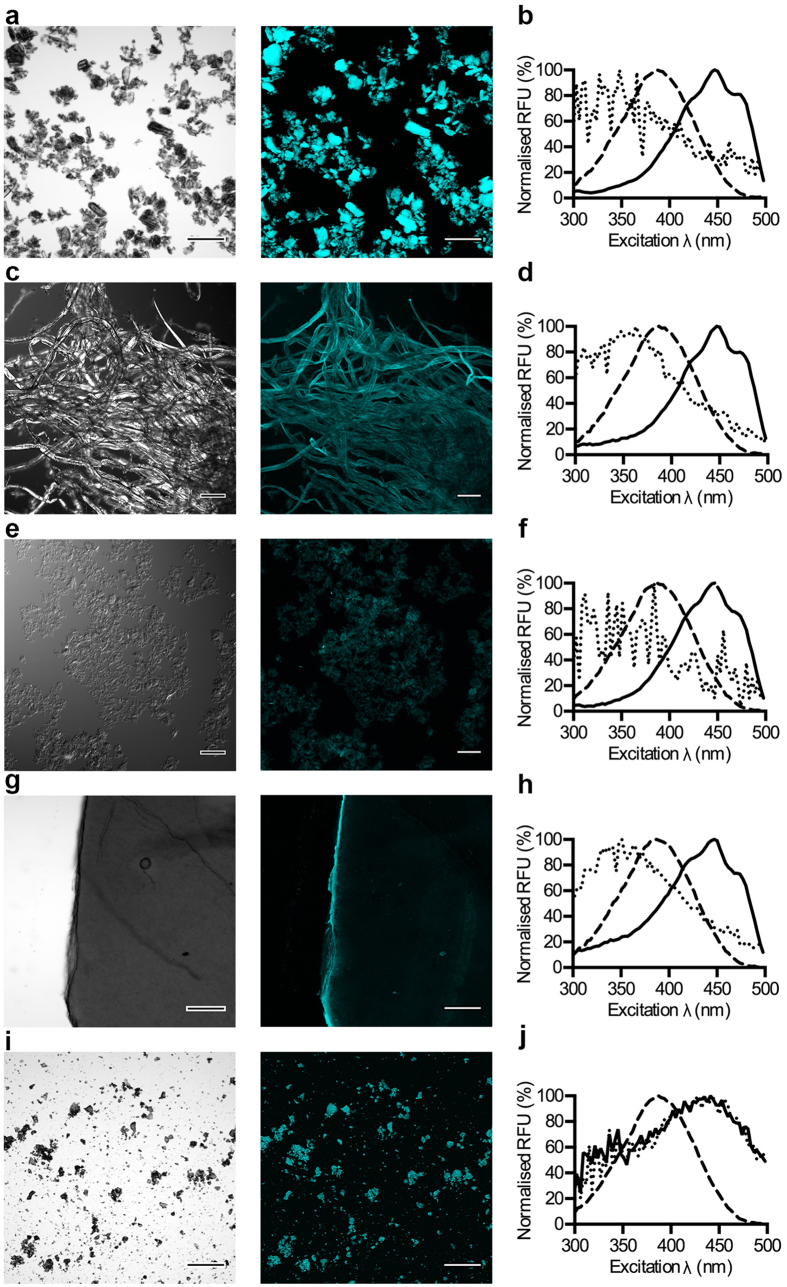
p-HTEA enables combined fluorescence imaging and spectrometric analysis of cellulose materials. Phase contrast (left) and fluorescence confocal microscopy (right) of (**a**) M. cellulose, (**c)** pulp cellulose, (**e**) cellulose nanofibrils, (**g**) paper made of cellulose nanofibrils, and (**i**) lignin stained with p-HTEA. In confocal microscopy, excitation at 473 nm and bandwidth filters detecting 490–530 nm were applied. Corresponding unstained controls are shown in Supplementary Fig. 2. Scale bar = 200 μm. Normalized excitation spectra of p-HTEA mixed with (**b**) M. cellulose, (**d**) pulp cellulose, (**f**) cellulose nanofibrils, (**h**) paper made of cellulose nanofibrils, and (**j**) lignin (solid). Spectra of the pure materials (dotted) and of p-HTEA (dashed) are shown for comparison.

The diverse structures adopted by cellulose-derived materials are traditionally visualized by methods such as electron microscopy. In addition, cellulose has also been visualized by fluorescence imaging using fluorophore labelled carbohydrate binding modules or small fluorescent molecules such as Congo red^34^,^35^. However, these conventional fluorophores do not display the binding induced spectral alterations as observed for p-HTEA. Taking advantage of the binding-specific optical features of p-HTEA, we next analysed if p-HTEA could be utilized for fluorescence-based microscopy, while at the same time serving as a means to analyse the material purity. Following immersion of the M. cellulose in p-HTEA, unbound probe was washed away before the material was mounted on a glass slide. Phase contrast microscopy revealed the highly crystalline nature of M. cellulose with a heterogeneous mix of crystal sizes (Fig. 5a). When studied by fluorescence confocal microscopy, p-HTEA bound to the microcrystalline cellulose fluoresced brightly, and the signal accurately co-localized with the position of crystals observed under phase contrast. No fluorescence signal was detected from M. cellulose incubated without p-HTEA (Supplementary Fig. 3a).

Subsequent to imaging, the excitation spectra of the materials were obtained by spectroscopy. The unique optical signature appeared only when p-HTEA and M. cellulose were co-incubated, thereby verifying the identity and purity of this cellulose material (Fig. 5b). Bleached pulp cellulose consists of a mesh of irregularly sized fibres, and staining with p-HTEA again revealed fluorescence signals that co-localized with the fibre mesh (Fig. 5c). In the absence of p-HTEA, this material showed a weak background fluorescence (Supplementary Fig. 3b). Analysis of the excitation spectrum suggests that this background signal originates from the presence of a chemical that excites in the violet region, whereas the prominent spectral signature of the sample mixed with p-HTEA discloses the purity of the bleached pulp cellulose (Fig. 5d).

Cellulose nanofibrils (CNF) showed a dispersed cloud-like appearance in both phase contrast and fluorescence microscopy when in the presence of p-HTEA, whereas no fluorescence was observed in the absence of the probe (Fig. 5e, Supplementary Fig. 3c). Analysis of the excitation spectra confirmed the cellulose nature of the material by the unique spectral signature (Fig. 5f). To generate a more dense structure of the CNF, we produced pieces of CNF paper. Phase contrast microscopy revealed its thick and compact appearance, with folds as well as air pockets (Fig. 5g). Fluorescent signal from p-HTEA bound to the material surface could be observed, whereas in the absence of p-HTEA, CNF paper was weakly fluorescent, particularly in the small pockets on the material (Supplementary Fig. 3d). This was confirmed by spectrometric analysis of the unstained material, whereas in the presence of p-HTEA, the unique cellulose excitation spectrum was easily recognized (Fig. 5h).

Finally, kraft lignin was analysed. It showed a powdery appearance under phase contrast, and displayed an inherent fluorescence regardless of the presence of p-HTEA (Fig. 5i, Supplementary Fig. 3e). Analysis of the associated excitation spectra suggested the presence of lignin (Fig. 5j). Collectively, our data shows the applicability of p-HTEA as an opto-tracer for direct morphological analysis and purity control of cellulose and lignin samples using fluorescence-based imaging and spectral analysis.

### Monitoring in real-time the enzymatic digestion of cellulose

**Figure 6:**
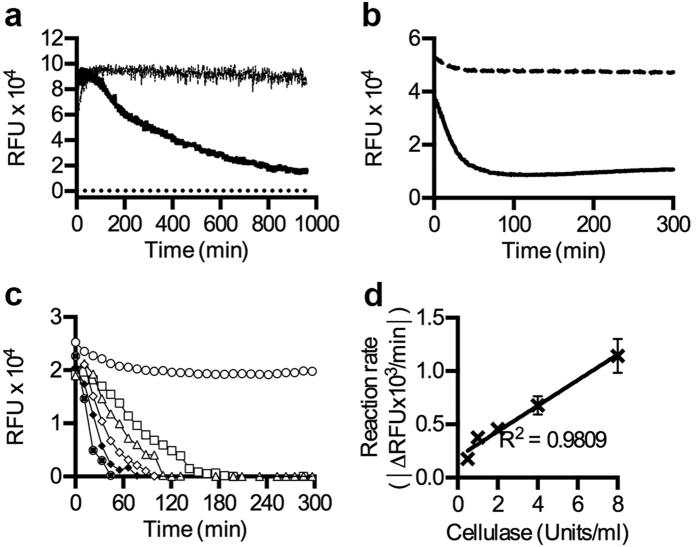
Cellulolysis recorded in real-time by fluorescence monitoring. (**a**) Fluorescence signal from p-HTEA mixed with M. cellulose in the presence (solid) and absence (dashed) of cellulase. Background signal is monitored in samples containing cellulase and p-HTEA, but no cellulose (dotted line). (**b**) Enzymatic digestion of paper made of cellulose nanofibrils. Decreased fluorescence occurs as cellulase digests the p-HTEA-stained paper (solid), whereas signal remains constant in the absence of enzyme (dashed). (**c**) Rapid degradation of p-HTEA-stained suspension of cellulose nanofibrils occurs when increasing units of cellulase (right to left 0, 0.5, 1, 2, 4, 8 U/ml) is added. (**d**) Linear regression analysis of each assay in (**c**) reveal linear correlation between reaction rate and enzyme units.

Cellulose-based bioethanol production is extensively explored as an alternative to petroleum in the search for sources of clean renewable energy^36^. Cellulolysis, which represents a key step in the production of cellulosic ethanol, is commonly performed by acid hydrolysis or enzymatic digestion, and the generated glucose is converted to ethanol via microbial fermentation^37^. To investigate the suitability of Oligothiophenes for real-time analysis of the efficiency of enzymatic processes, p-HTEA was applied to M. cellulose together with cellulase from *Trichoderma viride*. In contrast to the stable signal observed in the absence of cellulase, addition of the enzyme caused a gradual decrease in RFU over time (Fig. 6a). Additionally, we observed a relatively slow digestion rate (80.6 RFU/min) of M. cellulose. When assaying the digestion of a paper disk made of cellulose nanofibrils, the maximum rate of digestion occurred in the first 50 min, and reached equilibrium after 100 min (Fig. 6b). This demonstrates an approximately 10-fold increase of the reaction rate (746.5 RFU/min). The reaction rate was even further increased when cellulase digestion of a cellulose nanofibril suspension was assayed. Complete digestion occurred within 50 min, with a maximum rate of 1144 RFU/min (Fig. 6c). This indicates that the compact morphology of some sources of cellulose may hamper enzyme activity due to accessibility constraints. Importantly, our results showed that p-HTEA interacting with the cellulose chains does not affect enzyme activity. To assess the use of p-HTEA for real-time analysis of enzymatic digestion reactions, we next exposed suspensions of cellulose nanofibrils to different cellulase concentrations. Complete digestion occurred between 50–180 min depending on enzyme concentration. By performing a linear regression analysis, highly significant linear correlations between enzyme concentration and the rate of change of the fluorescence signal was seen (Fig. 6d). In contrast to current methods that measure cellulase activity based on end product generation of glucose^38^, p-HTEA provides a novel tool for direct real-time recording of cellulase activity based on its binding to the cellulose core β(1-4) D-glucose chain.

## Discussion

Based on the structure-responsive optical features of luminescent oligothiophenes, we have developed new, spectral-based methods for non-destructive determination and visualization of the chemical composition of lignocellulosic biomass. Having successfully shown the validity of the new methodology with defined polysaccharides and authentic samples from both sulphite and kraft pulping processes, the next step is up-scaling, automation, and integration in large-scale industrial biomass processes. Based on the simplicity of usage and data evaluation, the methods also have the potential to become primary tools for widespread application in academic research laboratories beyond those specialized in carbohydrate analysis, with a minimal requirement for specialized equipment or training. From a socio-economic perspective, selective quality assessment tools will aid in reducing the necessity of costly, complicated and chemical-consuming methods in bio-refineries where cellulosic biomass with complex matrix compositions are processed and fractionated. As new manufacturing processes built on the bio-refinery concept are implemented to meet society’s endeavour to develop biomass into renewable products and fuels, cellulose and related materials will continue to hold great scientific and economic value.

## Methods

### Oligothiophenes, cellulose, hemicelluloses, lignin and process liquors

p-HTEA, p-HTAA, p-FTAA and p-HTIm were synthesized as previously described^20^,^26^,^27^. The synthesis scheme of p-HTEA is shown in Supplementary Fig. 4. p-HTEA was first dissolved in DMSO to a concentration of 15 μM and then further diluted with ^D^H_2_O to a final concentration of 1.5 μM. The other oligothiophenes were dissolved directly in ^D^H_2_O to a final concentration of 1.5 mM. These stock solutions were maintained in the fridge. Unbleached and bleached cellulose was generously provided by Stora Enso, Skoghallsverken, Sweden. Xylan from beechwood (>90% xylose) and galactomannan from *Ceratonia siliqua* seeds (Locust bean gum) were from Sigma Aldrich. *O-*acetylgalactoglucomannan (degree of acetylation ∼0.30) from spruce was recovered from process liquor generated in thermomechanical pulping. The hemicellulose component was isolated by ultrafiltration and lyophilisation as previously described^39^. Process liquor withdrawn after pre-hydrolysis of hardwood was kindly provided by Södra Cell AB, Sweden. This liquor contains polymeric and oligomeric hemicellulose (>95% pure *O*-acetyl-4-*O*-methylglucuronoxylan) and ca 1% lignin^32^,^33^,^39^. Lignosulphonate, obtained from sulphite pulping, was generously provided by Domsjö Fabriker AB, Sweden. Kraft lignin, obtained from alkaline pulping, was purchased from Sigma-Aldrich, CAS no, 8068-05-01.

### Preparation of cellulose nanofibrils

Cellulose nanofibrils were prepared as previously described^25^ from bleached never-dried softwood pulp (Domsjö dissolving plus) containing 20% (w/w) dry content (~93% cellulose, ~5% hemicellulose) by 2,2,6,6-tetramethylpiperidine-1-oxyl radical (TEMPO) oxidation^1^,^2^. The oxidized pulp was defibrillated by mixing, ultrasonification and mechanical stirring until a gel of nanofibrils dispersed in water was achieved.

### Screening oligothiophenes against lignocellulosic materials

Serial dilutions of each carbohydrate were prepared in ^D^H_2_O ranging from 0.02–5 mg/ml. p-HTEA, p-HTAA, p-FTAA and p-HTIm were added at final concentrations of 3 μM in ^D^H_2_O in 1 ml aliquots of each carbohydrate dilution pertaining to Figs 1, 2 and 3. From each mix, 100 μl/well was dispensed into 96-well plates, each concentration assayed in triplicate. Using a Synergy MX plate reader (Biotek) excitation spectra were collected by exciting the sample from 300 to 500 nm and detecting emission at 545 nm. Positive detection of the carbohydrate was concluded when a change in wavelength of maximum excitation (λ_max_) and RFU intensity occurred with reference to the negative control. The correlation between signal intensity and carbohydrate concentration was shown by plotting the corresponding fluorescence signal of carbohydrate-bound oligothiophene against the concentration of each carbohydrate. The λ_Ex_ (emission detected at 545 nm) corresponds to either the λ_max_, or the excitation wavelength suffering the lowest background. Linear regression analysis is performed to determine the relationship between the RFU and carbohydrate concentration.

### Scanning electron microscopy

Topographical analysis of cellulosic materials was performed using a Hitachi s-4800 Field-Emission scanning electron microscope (FE-SEM) operating at 0.5 kV. The specimens were mounted on stubs using adhesive carbon tape and analysed without prior sputter coating. Electron micrographs of specimens were recorded at 500 x, except for nanofibrils, which were recorded at 5000 x due to their small size.

### Competitive binding assay between p-HTEA and carbohydrates

Preparations of each carbohydrate (1.25 mg/ml) mixed with p-HTEA (3 μM) in ^D^H_2_O were allowed to stand for 30 min. From each mixture, 50 μl aliquots were pipetted into individual wells of a 96 well plate. Solutions of the competing carbohydrates were prepared in ^D^H_2_O at concentrations ranging from 0.04–5 mg/ml. From each dilution, 50 μl was added to wells containing the pre-bound p-HTEA–carbohydrate mixture. Plates were analysed in a Synergy MX plate reader (Biotek) for the excitation spectrum of p-HTEA, as well as the emitted fluorescence at λ_Ex-max_, and emission at 530 or 540 nm.

### pH dependence of p-HTEA

1.25 mg/ml M. cellulose was prepared in 0.1 M sodium acetate (pH 4), phosphate buffered saline (PBS) (pH 7.4) and 0.1 M Na_2_HPO_4_ (pH 10). p-HTEA was added to an assay concentration of 4.5 μM. Emitted fluorescence was detected in a Synergy MX plate reader (Biotek) at λ_Ex_ = 445 nm, λ_Em_ = 530 nm.

### Visualization of cellulosic materials by phase contrast and fluorescence confocal microscopy

Materials (M. cellulose, pulp cellulose, cellulose nanofibrils, paper made of cellulose nanofibrils, lignin) were immersed in 1 ml staining solution containing 4.5 μM p-HTEA in ^D^H_2_O. Following 1 h incubation, samples were rinsed in 1 ml ^D^H_2_O, transferred to a glass slide, and imaged by phase contrast and fluorescence confocal microscopy using the 10 x objective on a Olympus FV1000 confocal microscope. p-HTEA bound to the materials was excited at 473 nm with fluorescence detected between 490 to 530 nm. Immediately after imaging, paper made of cellulose nanofibrils, and pulp cellulose were removed from the glass slide and placed in separate wells of a 96-well plate, each containing 100 μl ^D^H_2_O. Excitation spectrum for 300 nm to 500 nm was collected using the Synergy MX plate reader (Biotek) with emission detected at 545 nm. M. cellulose, lignin, and cellulose nanofibrils in suspension were analysed according to the previously described screening protocol.

### Enzymatic digestion of cellulosic materials

Aliquots of 100 μl of M. cellulose (1 mg/ml) and cellulose nanofibrils (0.29 mg/ml) mixed with p-HTEA (3 μM final concentration) in 50 mM sodium acetate buffer (pH 5) were positioned in the wells of 96-well plates. The cellulose nanofibril paper (5 mm disc, 3.5–6 mg) was positioned directly into wells and immersed by 100 μl of the p-HTEA solution. Immediately before the start of fluorescence recording, 100 μl cellulase from *Trichoderma viridae* (Sigma-Aldrich) was added to each well. 8 U cellulase was used except for when serial dilutions in 50 mM sodium acetate buffer (pH 5), ranging from 0–8 U, was used when titrations were performed. The 96-well plates were incubated under shaking conditions (medium setting) at 37 °C in a Synergy MX plate reader. Emitted fluorescence at Ex 480 nm and Em 530 nm for cellulose was measured in triplicate at 1 min interval.

## Additional Information

**How to cite this article**: Choong, F. X. *et al*. Nondestructive, real-time determination and visualization of cellulose, hemicellulose and lignin by luminescent oligothiophenes. *Sci. Rep.* **6**, 35578; doi: 10.1038/srep35578 (2016).

## Supplementary Material

Supplementary Information
