# Supplementary material for: Nondestructive, real-time determination and visualization of cellulose, hemicellulose and lignin by luminescent oligothiophenes
Source: Sci Rep. 2016 Oct 19;6:35578. doi: 10.1038/srep35578 (PMC5069672; doi:10.1038/srep35578)
Supplement: Supplementary Information [file srep35578-s1.pdf]

SUPPLEMENTARY INFORMATION

Nondestructive, real-time determination and visualization of cellulose, hemicellulose and lignin by luminescent oligothiophenes

Ferdinand X. Choong[1], Marcus Bäck[2], Svava E. Steiner[1], Keira Melican[1], K. Peter R. Nilsson[2], Ulrica Edlund[3], Agneta Richter-Dahlfors[1*]

[1] Swedish Medical Nanoscience Center, Department of Neuroscience, Karolinska Institutet, Stockholm, SE-171 77, Sweden.

[2] Division of Chemistry, Department of Physics, Chemistry and Biology, Linköping University, Linköping, SE-581 83, Sweden

[3] Fiber and Polymer Technology, KTH Royal Institute of Technology, Stockholm, SE-100 44, Sweden.

Supplementary Figure 1

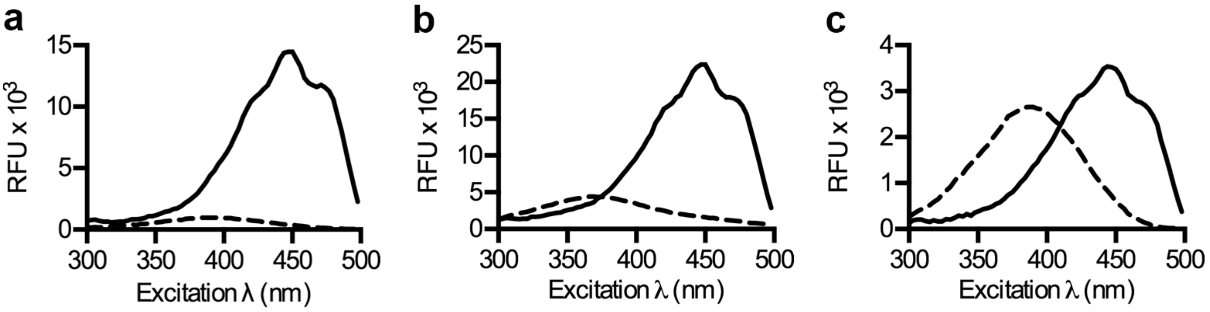

Binding of pHTEA to cellulose results in increased amplitude of the fluorescence signal.

Excitation spectra of pHTEA bound to the cellulosic materials. Spectra were collected at λ_{Ex} = 300 – 500 nm and λ_{Em} = 545 nm of pHTEA mixed with (**a**) M. cellulose, (**b**) pulp cellulose, and (**c**) cellulose nanofibrils (solid lines). RFU = Relative fluorescence unit. Dashed line = pHTEA in $^{D}H_2O$.

Supplementary Figure 2

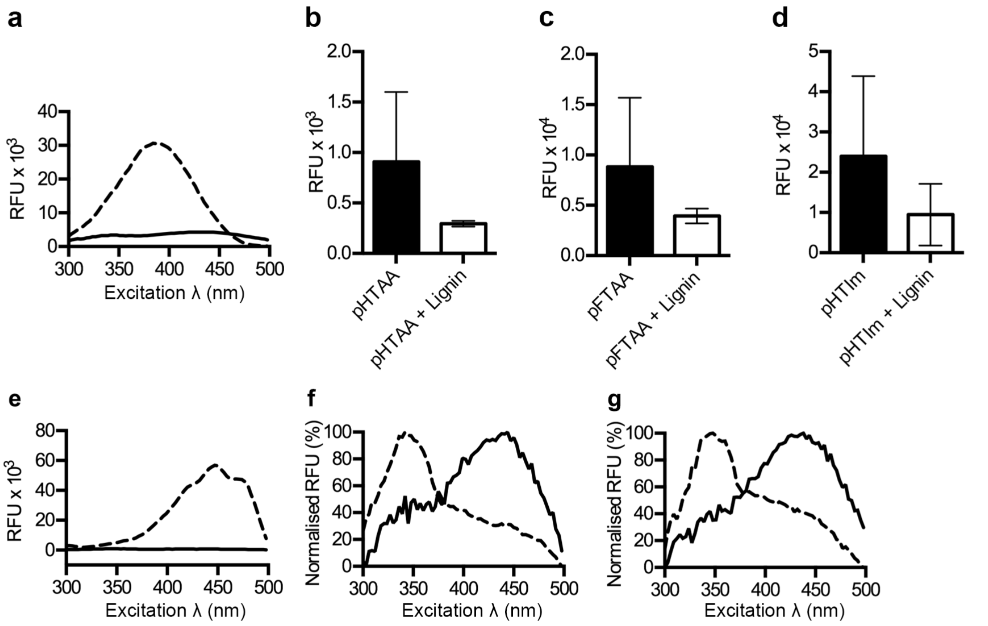

Binding of pHTEA to lignin enables optical identification of lignin in mixed samples.

(**a**) Excitation spectra of pHTEA with 0 (dashed) and 5 mg/ml lignin (solid) collected at λ_{Ex} = 300 – 500 nm, λ_{Em} = 545 nm. (**b**) Comparison of fluorescence signals at $\lambda_{max\text{-}pHTEA}$ (λ_{Ex} 387 nm, λ_{Em} 545 nm) of pHTAA, (**c**) pFTAA and (**d**) pHTIm in the presence and absence of 5 mg/ml of lignin. (**e**) Excitation spectra of pHTEA bound to 1.25 mg/ml cellulose before (dashed) and after (solid) addition of 5 mg/ml lignin. (**f**) Normalised excitation spectra of pHTEA + 1.25 mg/ml lignin (dashed) and pHTEA + 1.25 mg/ml lignin + 5 mg/ml cellulose (solid). (**g**) Normalised excitation spectra of intrinsic fluorescence of 1.25 mg/ml lignin (dashed) and 1.25 mg/ml lignin + 5 mg/ml cellulose (solid). All panels show the mean of three technical repeats from one out of three experimental repeats. RFU = Relative fluorescence unit. All experiments are performed in $^{D}H_2O$, pHTEA is used at 3 μM.

Supplementary Figure 3

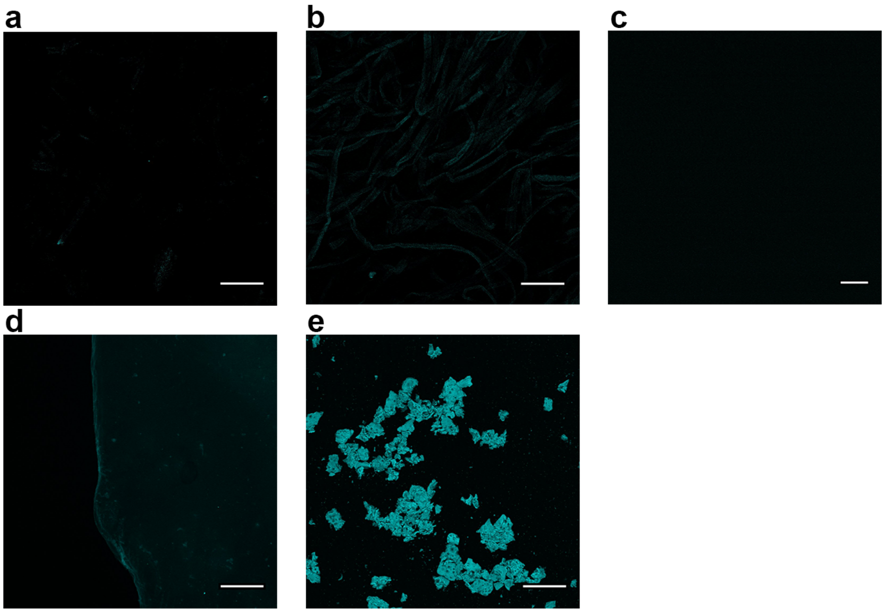

Fluorescence of cellulose materials analyzed by confocal microscopy.

Fluorescence confocal microscopy showing the inherent fluorescence of (**a**) M. cellulose, (**b**) pulp cellulose, (**c**) cellulose nanofibrils, (**d**) paper made of cellulose nanofibrils, and (**e**) lignin. Excitation at 473 nm and bandwidth filters detecting 490 - 530 nm were applied. Scale bar = 200 μm.

Supplementary Figure 4

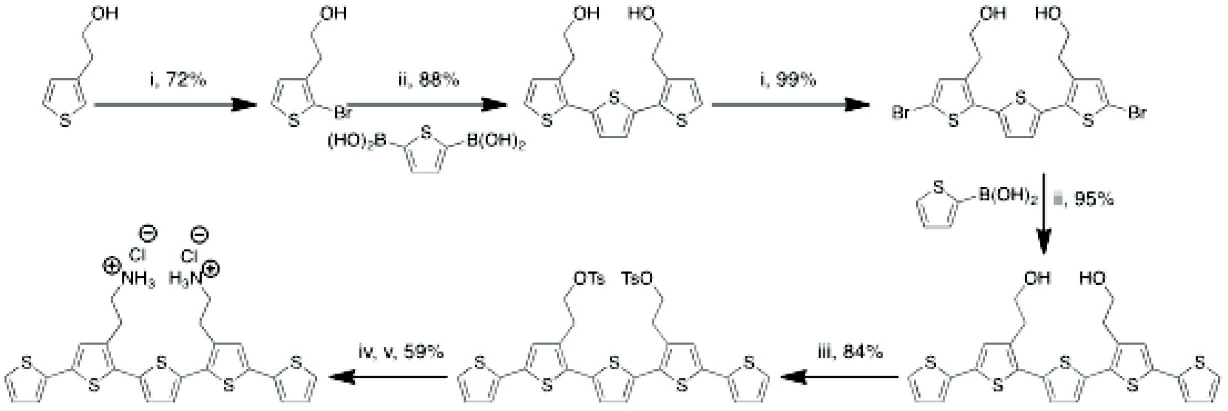

Schematic illustration of the pHTEA synthesis scheme.

Reagents and conditions are as follows: (i) N-bromosuccinimide, DMF, -10°C; (ii) PEPPSI-IPr, K_2CO_3, toluene/methanol (1:1), 75°C; (iii) p-toluenesulphonyl chloride, pyridine, $CHCl_3$; (iv) $NHBoc_2$, DMF, 75°C; (v) HCl (conc), dioxane. Numbers in % denotes the overall yield. Full description of the synthesis is found in Reference 26 and 27.
